# External Jugular Vein Aneurysm Presenting as a Cervical Mass

**DOI:** 10.1155/2011/485293

**Published:** 2011-05-16

**Authors:** Eleni E. Drakonaki, Emmanouil K. Symvoulakis, Anthoula Fachouridi, Dimitrios Kounalakis, Emmanouil Tsafantakis

**Affiliations:** ^1^Department of Radiology, Venizeleion General Hospital, 71409 Heraklion, Crete, Greece; ^2^Primary Care Unit, Clinic of Social and Family Medicine, Faculty of Medicine, University of Crete, 71003 Hraklion, Crete, Greece; ^3^Anogia Primary Care Unit, 70012 Heraklion, Crete, Greece

## Abstract

Venous aneurysms are rare causes of neck mass. Among neck veins, aneurysms of the external jugular vein are extremely uncommon. We present a case of a woman with a history of prior internal jugular vein catheterization who presented at a rural primary health care unit with a nontender progressively enlarging swelling in the right supraclavicular region. B-mode and Doppler ultrasound examination revealed a saccular dilatation of the external jugular vein, suggesting a posttraumatic venous aneurysm. Saccular aneurysms of the external jugular vein are uncommon and only rarely lead to serious complications. Access to ultrasound examination can allow early detection of this entity.

## 1. Introduction

Venous aneurysms are a rare clinical entity [1–3] and their natural history depends on their anatomic location [2]. Those in the head and neck region usually have a benign clinical course causing only pain and tenderness, as opposed to those at other locations that may lead to embolism or rupture [2]. The diagnosis may be suggested by clinical features and is usually confirmed by imaging. Although multidetector computed tomography (MDCT) angiography or selective venography allows for accurate diagnosis, ultrasonography (US) with colour Doppler imaging is the gold standard for the diagnosis of such aneurysms [4, 5].

Venous aneurysms have been reported in several anatomic locations in the neck, the commonest site being the internal jugular vein [3, 4]. Although fusiform cervical venous dilatations represent a frequent occurrence, saccular venous aneurysms of the external jugular veins are extremely rare, with a few cases been reported in the English literature, all involving true venous aneurysms and none associated with prior venous catheterization [5–10]. We report a case of a 74-year-old woman with a saccular aneurysm of the external jugular vein possibly associated with prior internal jugular vein catheterization, which was diagnosed using Doppler US and confirmed by MDCT angiography.

## 2. Report of a Case

**Figure 1:**
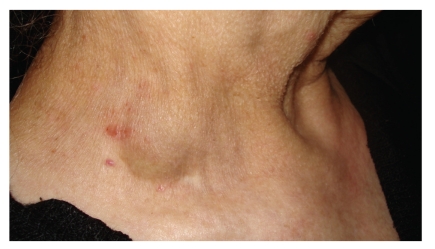
A 74-year-old woman presented with a lump at the right supraclavicular fossa which had been enlarging progressively over a period of a few months. The lesion corresponded to an aneurysm of the external jugular vein.

A 74-year-old woman presented to the general practitioner at a primary health care unit with a swelling in the right supraclavicular region which had been enlarging progressively over a period of a few months. Physical examination revealed a soft, nontender, nonpulsative lump at the right supraclavicular region (Figure 1). The skin overlying the mass had no signs of inflammation. The lesion was slightly enlarging with Valsalva maneuver. History revealed hospitalization in the intensive care unit due to a viral encephalopathy two years earlier, when internal jugular vein catheterization had been performed with no immediate complications. 

**Figure 2:**
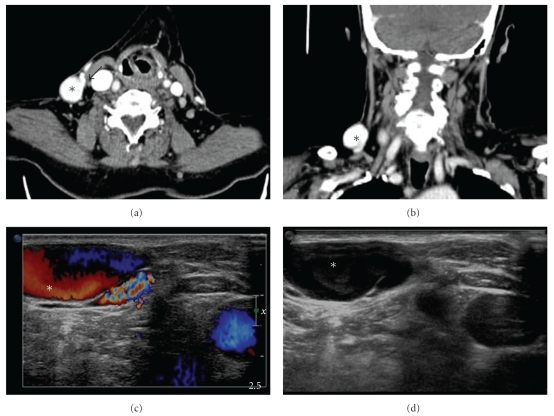
Axial (a) and (b) coronal Multidetector Computed Tomography (MDCT) images following intravenous contrast administration and color Doppler (c) and gray scale (d) ultrasound images of an external jugular vein aneurysm. Ultrasound and MDCT showed the presence of a cystic mass (asterisks) communicating with a short neck (arrow) with the external jugular vein. The lesion presented with venous flow on colour Doppler US examination and intense contrast enhancement on MDCT.

Chest, abdomen examination, and blood tests were unremarkable. After a first US examination within the primary care facility, the patient was referred for a confirmatory US evaluation from a qualified radiologist, which revealed a 2.2 cm cystic mass which increased in size with Valsalva maneuver, was completely compressible, and communicated with a short neck with the adjacent right external jugular vein. The lesion exhibited slow internal flow in B-mode and aliasing in Doppler imaging. Spectral Doppler analysis showed venous waveform. The findings were suggestive of a saccular aneurysm of the external jugular vein with no evidence of thrombosis (Figure 2). Multidetector computed tomography (MDCT) angiography confirmed the presence of an enhancing oval-shaped noncalcified saccular lesion at the anterior wall of the external jugular vein, in keeping with an external jugular venous aneurysm. MDCT also showed the short neck of the aneurysm. The right internal jugular vein was normal.

The patient was referred to the vascular surgery department of our hospital but denied surgical treatment. Follow-up US examination 6 months later revealed no substantial changes.

## 3. Discussion

Acquired venous aneurysms can be the result of several processes including tumors, inflammation, trauma or can appear spontaneously, when no etiologic cause can be identified [11, 12]. They are saccular, as opposed to the congenital fusiform dilation seen in children [12, 13]. In the present case, this was a saccular aneurysm which appeared spontaneously. Although the exact aetiology remains unknown, it is likely of iatrogenic origin. To the best of our knowledge, this case of external jugular vein aneurysm is the first to be reported in the literature as an iatrogenic complication resulting from prior percutaneous internal jugular vein catheterization. The most common vascular lesions after failing to locate the internal jugular vein are reported to be pseudoaneurysms [14]. We assume that the attempt for catheterization of the internal jugular vein had likely led to trauma at the adjacent external jugular vein, resulting in weakening of the venous wall and aneurysmatic dilatation of the vein at a later stage. Although the catheterization procedure had been reported as uneventful and no immediate complications were reported in the patient's notes, the procedure had been performed without sonographic guidance and therefore trauma at the adjacent structures would have been highly probable. As venous pressure is not high enough to install immediate ectatic phenomena [7, 15], venous dilatation presented as a long-term iatrogenic complication. Although it is suggested that major embolic complications due to jugular vein aneurysms are unlikely to be expected [12, 16, 17], this cannot be marginalized since the low incidence of cervical venous aneurysms does not allow the study of large cases series [16]. Moreover, a case of a thrombosed external venous aneurysm causing undetected pulmonary embolisms has recently been reported, showing that those aneurysms may not be as innocent as previously thought [9].

In the clinical setting, the differential diagnosis of a neck mass in an adult usually includes lymph node enlargement, carcinomas of adjacent organs, laryngocele, and a variety of cystic formations [12]. Venous aneurysms are rarely included, especially when no neck trauma is reported in history. Although the diagnosis can be occasionally suspected on clinical signs, ultrasonography is the method of choice for the evaluation of a neck mass. Ultrasonography can easily differentiate between cystic and solid lesions, establish the origin of the lesion from adjacent structures, differentiate vascular from nonvascular lesions using colour Doppler imaging and depict the characteristic imaging features which will allow for a specific diagnosis. Cysts and laryngoceles can be easily differentiated from venous lesions, as there is neither flow on colour Doppler ultrasonography nor communication within the vascular system in such cases [6, 18]. Among vascular lesions, arterial pseudoaneurysms and traumatic arteriovenous fistulas can be easily differentiated from venous aneurysms using Doppler ultrasonography by depicting the characteristic arterial or turbulent waveform and the communication with an artery or between arterial and venous segments, respectively [18]. 

In our case the diagnosis was suggested from a rural primary care setting and subsequently confirmed by qualified radiologists. Nowadays, the use of portable US devices has made US increasingly available to physicians, allowing for the imaging work-up to be performed even in primary care establishments [18]. Thus, clinical awareness of the differential diagnosis together with US evaluation of a neck mass within primary care settings may trigger the interface between primary and secondary care and allow early diagnosis to improve diagnostic outcomes.

## 4. Conclusions

Acquired venous aneurysms of the external jugular vein are rare and can present spontaneously in adult patients after percutaneous catheterization of the internal jugular vein. Such lesions are likely to present within primary care and ultrasound can aid in the diagnosis since it may be performed in these settings due to its low cost, repeatability, and good diagnostic accuracy. Reporting this case of a venous aneurysm of the jugular vein system, we showed that such lesions can potentially challenge the diagnostic capacity of the involved physicians.
